# Supplementary material for: In Vivo Metabolism of [1,6-13C2]Glucose Reveals Distinct Neuroenergetic Functionality between Mouse Hippocampus and Hypothalamus
Source: Metabolites. 2021 Jan 12;11(1):50. doi: 10.3390/metabo11010050 (PMC7828183; doi:10.3390/metabo11010050)
Supplement: Supplementary file 1 [file metabolites-11-00050-s001.zip › Cherix_et_al-Supplementary_material.pdf]

Supplementary Figures

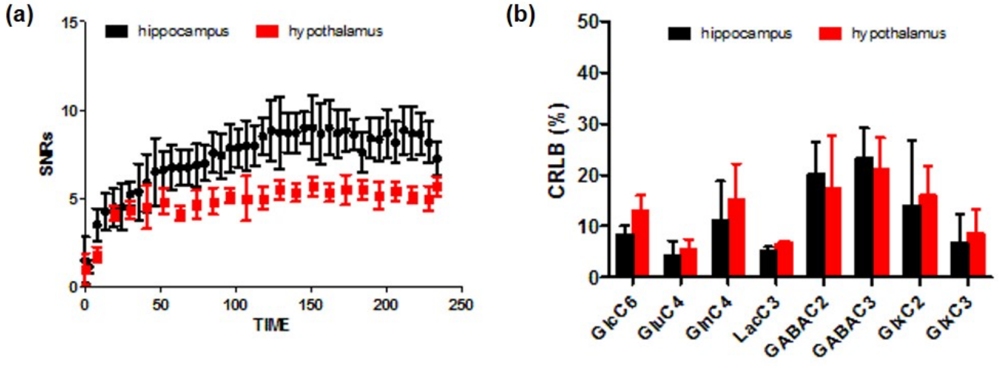

<u>Supplementary Figure 1:</u> Spectra quality comparison

Overall SNRs **(a)** of the *edited* spectra and the corresponding CRLBs **(b)** of major ^{13}C-metabolites from hippocampus (black) and hypothalamus (red). Data are presented as mean±SD. Abbreviations: GlcC6, glucose C6; GluC4, glutamate C4; GlnC4, glutamine C4; LacC3, lactate C3; GABAC2, γ-aminobutyric acid C2; GABAC3, γ-aminobutyric acid C3; GlxC2, glutamate C2 + glutamine C2; GlxC3, glutamate C3 + glutamine C3.

Brain region	Infusion time (min)	LacC3	GluC4	GlnC4	GABAC2	GABAC3	GlxC3	GlxC2
hippocampus	*5*							
	15	0.51±0.02						
	90							
	100-130		0.50 ±0.01	0.46 ±0.02 (***)				
	150				0.42 ±0.03		0.42±0.02 (*)	0.35±0.02
	200					0.38 ±0.03		
hypothalamus	*5*							
	15	0.51±0.02						
	90							
	100-130		0.51 ±0.01	0.52 ±0.02				
	150				0.43 ±0.03		0.46±0.03	0.34±0.03
	200					0.36 ±0.03		

<u>Supplementary Table 1:</u> Metabolite fractional enrichment comparison

Isotopic fractional enrichments (FEs) of ^{13}C-labeled resonances at their respective time to reach steady-state in hippocampus and hypothalamus. Data are presented as mean±SD. Statistical significance between hippocampus and hypothalamus was obtained from unpaired Student's t-test with *$p<0.05$ and ***$p<0.001$.

Energy yielding pathway	Flux	ATP yield	Hippocampus (µmol/g/min)	ATP (µmol/g/ min)	Hypothalamus (µmol/g/min)	ATP (µmol/g/ min)
**Glycolysis**	CMR_{Glc}	**2**	0.29	0.58	0.47	0.94
**Krebs cycle**	$V_{TCA} + V_{GABA} + V_{PC}$ ($=CMR_{Glc}(ox)$)	**~ 23**	0.81	18.7	0.97	22.3
**Cytoplasmic NADH production**	$2 * CMR_{Glc} + V_{dil}^{in} - V_{dil}^{out}$		0.60		0.64	
**NADH entering MAS**	V_x	**~2.5 ATP/ NADH**	0.50	1.25	0.20	0.50
**Remaining cytoplasmic NADH**	$2 * CMR_{Glc} + V_{dil}^{in} - V_{dil}^{out} - V_x$		**0.10**		**0.44**	
**TOTAL ATP production**				**20.5**		**23.7**

<u>Supplementary Table 2:</u> Summary of hippocampal and hypothalamic energy consumption calculated from the estimated metabolic fluxes *in vivo*.

The ATP yields (from Hertz et al.[1] for each energy pathway were multiplied by the given fluxes in µmol/g/min to obtain the ATP production. Consumption of one molecule of glucose produces 2 ATP and 2 NADH. Mitochondrial function produces ~23 ATP from the action of pyruvate dehydrogenase (PDH) and tricarboxylic acid (TCA) per pyruvate molecule (referred to as 'Krebs cycle' here). The NADH produced through glycolysis can be recycled by forming lactate from pyruvate or shuttled in the mitochondria for oxidation in the electron transport chain (ETC) through the malate-aspartate shuttle (MAS), yielding ~2.5 ATP per molecule of NADH. The remaining cytoplasmic NADH, i.e. the difference between the net NADH produced from glycolysis or blood lactate influx and MAS flux (V_x), corresponds to the proportion of NADH that is not oxidized and might serve a biosynthetic purpose.

Mathematical description of 1-compartment model of brain glucose metabolism

The model presented in Figure 3 is an extension of the model presented in Lizarbe et al.[2] supplemented by feature from Cherix et al.[3]. This model is described with mass-balance and isotope balance equations, assuming a steady-state condition and metabolic pool sizes to be constant over the time of [1,6-$^{13}C_2$]Glc infusion. Metabolite concentration was measured from ^{1}H-MRS neurochemical profile and ^{13}C-isotopic fractional enrichment (FE) from ^{1}H-[^{13}C]-MRS.

Assumptions

Glucose fuels glycolytic and mitochondrial metabolism in two consecutive paths. Firstly, glucose is metabolized through glycolysis, or more specifically the cerebral metabolic rate of glucose (CMR_{Glc}), to produce pyruvate in a non-oxidative way. Then, pyruvate is oxidized in the mitochondria through the action of pyruvate dehydrogenase (PDH). To include the part of pyruvate that does not arise from glycolysis but exchanges with blood lactate, the net brain lactate influx (${V_{dil}}^{in} - {V_{dil}}^{out}$) has to be considered as well:

$$V_{PDH} = 2 \cdot CMR_{Glc} + {V_{dil}}^{in} - {V_{dil}}^{out} = V_{TCA}$$

Brain and blood lactate relation was set as previously described in Cherix et al.[3], by defining the net lactate influx and efflux as proportional to the respective concentrations:

$${V_{dil}}^{out} = {V_{dil}}^{in} \frac{Lac_{brain}}{Lac_{blood}}$$

Plasma ^{13}C-labelling of acetate and lactate, arising from peripheral metabolism of [1,6-$^{13}C_2$]Glc, was considered as described previously[3] by including the final FE values measured herein. Interpolation of plasma lactate data was included in the model with a final FE plateau set at 0.5 (at t_4=250 min). As we did not detect acetate with our current protocol, the final FE was set as descried in Cherix et al.[3].

$$\frac{Lac_3(t=250)_{Blood}}{Lac_{Blood}} = 0.5 \qquad \frac{Acetate_2(t=250)_{Blood}}{Acetate_{Blood}} = 0.5$$

Pyruvate was assumed to be in fast exchange with lactate, and both FE were thus considered to be equal.[4,5]

$$\frac{Pyr_3(t)}{Pyr} = \frac{Lac_3(t)}{Lac}$$

Set of differential equations

The set of differential equations used in the mathematical model is shown below. The small intermediates (OAA, OG and AcCoA) pool sizes were all approximated at 0.1 μmol/g as described in Cherix et al.[3]. The mathematical model used the brain glucose fractional enrichment as input function (IF). The estimated fluxes were: The cerebral metabolic rate of glucose (CMR_{Glc}), a dilution flux from plasma lactate (${V_{dil}}^{in}$), the tricarboxylic acid cycle flux ($V_{TCA} = 2 \cdot CMR_{Glc}(ox)$), transmitochondrial flux (V_x), the neurotransmission or Gln/Glu cycle flux (V_{NT}), and a dilution factor that accounts for glial metabolism that fuels oxidation in the mitochondria such as glycogen metabolism or oxidation of ketone bodies, fatty acids or acetate (${V_{dil}}^{g}$).[6] GABA is synthesized from glutamate through the action of glutamate decarboxylase (V_{GABA}) and was assumed to be localized in two compartments in exchange, as a result of the action of both GAD isoforms (GAD65 and GAD67), leading to its dilution (${V_{ex}}^{i}$).[3] The two pools were assumed to be equal in both regions based on relative GAD distribution.[7]

Glu C4: $$\frac{dGlu_4}{dt} = (V_x + V_{GABA} + V_{eff}) \cdot \frac{OG_4(t)}{OG} + V_{NT} \cdot \frac{Gln_4(t)}{Gln} - (V_x + V_{NT} + V_{GABA} + V_{eff}) \cdot \frac{Glu_4(t)}{Glu}$$

Glu C3: $$\frac{dGlu_3}{dt} = (V_x + V_{GABA} + V_{eff}) \cdot \frac{OG_3(t)}{OG} + V_{NT} \cdot \frac{Gln_3(t)}{Gln} - (V_x + V_{NT} + V_{GABA} + V_{eff}) \cdot \frac{Glu_3(t)}{Glu}$$

Glu C2: $$\frac{dGlu_2}{dt} = (V_x + V_{GABA} + V_{eff}) \cdot \frac{OG_2(t)}{OG} + V_{NT} \cdot \frac{Gln_2(t)}{Gln} - (V_x + V_{NT} + V_{GABA} + V_{eff}) \cdot \frac{Glu_2(t)}{Glu}$$

Gln C4: $$\frac{dGln_4}{dt} = (V_{NT} + V_{eff}) \cdot \frac{Glu_4(t)}{Glu} - (V_{NT} + V_{eff}) \cdot \frac{Gln_4(t)}{Gln}$$

Gln C3: $$\frac{dGln_3}{dt} = (V_{NT} + V_{eff}) \cdot \frac{Glu_3(t)}{Glu} - (V_{NT} + V_{eff}) \cdot \frac{Gln_3(t)}{Gln}$$

Gln C2: $$\frac{dGln_2}{dt} = (V_{NT} + V_{eff}) \cdot \frac{Glu_2(t)}{Glu} - (V_{NT} + V_{eff}) \cdot \frac{Gln_2(t)}{Gln}$$

Asp C3: $$\frac{dAsp_3}{dt} = V_x \cdot \frac{OAA_3(t)}{OAA} - V_x \cdot \frac{Asp_3(t)}{Asp}$$

Asp C2: $$\frac{dAsp_2}{dt} = V_x \cdot \frac{OAA_2(t)}{OAA} - V_x \cdot \frac{Asp_2(t)}{Asp}$$

OG C4: $$\frac{dOG_4}{dt} = (V_{TCA} + V_{GABA} + V_{PC}) \cdot \frac{AcCoA_2(t)}{AcCoA} + V_x \cdot \frac{Glu_4(t)}{Glu} - (V_x + V_{TCA} + V_{GABA} + V_{PC}) \cdot \frac{OG_4(t)}{OG}$$

OG C3: $$\frac{dOG_3}{dt} = (V_{TCA} + V_{GABA} + V_{PC}) \cdot \frac{OAA_2(t)}{OAA} + V_x \cdot \frac{Glu_3(t)}{Glu} - (V_x + V_{TCA} + V_{GABA} + V_{PC}) \cdot \frac{OG_3(t)}{OG}$$

OG C2: $$\frac{dOG_2}{dt} = (V_{TCA} + V_{GABA} + V_{PC}) \cdot \frac{OAA_3(t)}{OAA} + V_x \cdot \frac{Glu_2(t)}{Glu} - (V_x + V_{TCA} + V_{GABA} + V_{PC}) \cdot \frac{OG_2(t)}{OG}$$

OAA C3: $\frac{dOAA_3}{dt} = V_{PC} \cdot \frac{Pyr_3(t)}{Pyr} + \frac{1}{2} \cdot V_{TCA} \cdot \frac{OG_4(t)}{OG} + \frac{1}{2} \cdot V_{TCA} \cdot \frac{OG_3(t)}{OG} + V_x \cdot \frac{Asp_3(t)}{Asp} - (V_x + V_{TCA} + V_{GABA} + V_{PC}) \cdot \frac{OAA_3(t)}{OAA}$

OAA C2: $\frac{dOAA_2}{dt} = V_{PC} \cdot 0.011 + \frac{1}{2} \cdot V_{TCA} \cdot \frac{OG_4(t)}{OG} + \frac{1}{2} \cdot V_{TCA} \cdot \frac{OG_3(t)}{OG} + V_x \cdot \frac{Asp_2(t)}{Asp} - (V_x + V_{TCA} + V_{GABA} + V_{PC}) \cdot \frac{OAA_2(t)}{OAA}$

Lac C3: $\frac{dLac_3}{dt} = 2 \cdot CMR_{Glc} \cdot IF + {V_{dil}}^{in} \cdot \frac{Lac_3(t)_{Blood}}{Lac_{Blood}} - (V_{TCA} + {V_{dil}}^{out}) \cdot \frac{Lac_3(t)}{Lac}$

AcCoA C2: $\frac{dAcCoA_2}{dt} = V_{TCA} \cdot \frac{Pyr_3(t)}{Pyr} + {V_{dil}}^{g} \cdot \frac{Acetate_2(t)_{Blood}}{Acetate_{Blood}} - (V_{TCA} + {V_{dil}}^{g} + V_{GABA} + V_{PC}) \cdot \frac{AcCoA_2(t)}{AcCoA}$

GABA C2 (pool1): $\frac{d{GABA_2}^1}{dt} = V_{GABA} \cdot \frac{Glu_4(t)}{Glu} + {V_{ex}}^{i} \cdot \frac{{GABA_2}^2(t)}{GABA^2} - (V_{GABA} + {V_{ex}}^{i}) \cdot \frac{{GABA_2}^1(t)}{GABA^1}$

GABA C3 (pool1): $\frac{d{GABA_3}^1}{dt} = V_{GABA} \cdot \frac{Glu_3(t)}{Glu} + {V_{ex}}^{i} \cdot \frac{{GABA_3}^2(t)}{GABA^2} - (V_{GABA} + {V_{ex}}^{i}) \cdot \frac{{GABA_3}^1(t)}{GABA^1}$

GABA C4 (pool1): $\frac{d{GABA_4}^1}{dt} = V_{GABA} \cdot \frac{Glu_2(t)}{Glu} + {V_{ex}}^{i} \cdot \frac{{GABA_4}^2(t)}{GABA^2} - (V_{GABA} + {V_{ex}}^{i}) \cdot \frac{{GABA_4}^1(t)}{GABA^1}$

GABA (pool 2): $\frac{d{GABA_x}^2}{dt} = {V_{ex}}^{i} \cdot \left(\frac{{GABA_x}^1(t)}{GABA^1} - \frac{{GABA_x}^2(t)}{GABA^2}\right)$

References

1. Hertz L, Peng L, Dienel GA. Energy Metabolism in Astrocytes: High Rate of Oxidative Metabolism and Spatiotemporal Dependence on Glycolysis/Glycogenolysis. *J Cereb Blood Flow Metab* 2007; 27: 219–249.
2. Lizarbe B, Lei H, Duarte JMN, et al. Feasibility of in vivo measurement of glucose metabolism in the mouse hypothalamus by 1H-[13C] MRS at 14.1T. *Magn Reson Med* 2018; 80: 874–884.
3. Cherix A, Donati G, Lizarbe B, et al. Excitatory/inhibitory neuronal metabolic balance in mouse hippocampus upon infusion of [U- 13 C 6]glucose. *J Cereb Blood Flow Metab* 2020; 632: 0271678X2091053.
4. Leong SF, Lai JC, Lim L, et al. Energy-metabolizing enzymes in brain regions of adult and aging rats. *J Neurochem* 1981; 37: 1548–56.
5. Xu S, Yang J, Shen J. In vivo13C saturation transfer effect of the lactate dehydrogenase reaction. *Magn Reson Med* 2007; 57: 258–264.
6. Duarte JMN, Lanz B, Gruetter R. Compartmentalized cerebral metabolism of [1,6- 13C]glucose determined by in vivo 13C NMR spectroscopy at 14.1 T. *Front Neuroenergetics* 2011; 3: 1–15.
7. Sheikh S., Martin S., Martin D. Regional distribution and relative amounts of glutamate decarboxylase isoforms in rat and mouse brain. *Neurochem Int* 1999; 35: 73–80.
